# CHF6523 data suggest that the phosphoinositide 3-kinase delta isoform is not a suitable target for the management of COPD

**DOI:** 10.1186/s12931-024-02999-5

**Published:** 2024-10-19

**Authors:** Mirco Govoni, Michele Bassi, Luca Girardello, Germano Lucci, François Rony, Rémi Charretier, Dmitry Galkin, Maria Laura Faietti, Barbara Pioselli, Gloria Modafferi, Rui Benfeitas, Martina Bonatti, Daniela Miglietta, Jonathan Clark, Frauke Pedersen, Anne-Marie Kirsten, Kai-Michael Beeh, Oliver Kornmann, Stephanie Korn, Andrea Ludwig-Sengpiel, Henrik Watz

**Affiliations:** 1 Chiesi Farmaceutici SpA, Parma, Italy; 2 Alira Health Srl, Milan, Italy; 3 Chiesi SAS, Bois Colombes, France; 4 Chiesi USA, Inc, Cary, NC USA; 5 Chiesi Pharma AB, Stockholm, Sweden; 6 Biological Chemistry, Babraham Institute, Cambridge, UK; 7 Velocity Clinical Research Grosshansdorf, Formerly Known as Pulmonary Research Institute at LungenClinic Grosshansdorf, Airway Research Center North (ARCN), Member of the German Center for Lung Research (DZL), Grosshansdorf, Germany; 8 insaf Respiratory Research Institute, Wiesbaden, Germany; 9 IKF Pneumologie Frankfurt, Clinical Research Centre Respiratory Diseases, Frankfurt, Germany; 10 IKF Pneumologie Mainz, Mainz, Germany; 11 Thoraxklinik Heidelberg, Heidelberg, Germany; 12 Velocity Clinical Research Lübeck GmbH, Formerly Known as KLB Health Research, Lübeck, Germany; 13 Present Address: Respiratory Medicine Unit, Department of Medicine Solna and Center for Molecular Medicine, Karolinska Institutet, Stockholm, Sweden

**Keywords:** Phosphatidylinositol 3-kinases, Therapeutics, Proteomics, Gene expression profiling, Multi-omics

## Abstract

**Background:**

Chronic obstructive pulmonary disease (COPD) is a chronic inflammatory condition. Given patients with COPD continue to experience exacerbations despite the availability of effective therapies, anti-inflammatory treatments targeting novel pathways are needed. Kinases, notably the phosphoinositide 3-kinases (PI3K), are thought to be involved in chronic airway inflammation, with this pathway proposed as a critical regulator of inflammation and oxidative stress response in COPD. CHF6523 is an inhaled PI3Kδ inhibitor that has shown positive preclinical results. This manuscript reports the results of a study of CHF6523 in patients with stable COPD (chronic bronchitis phenotype), and who had evidence of type-2 inflammation.

**Methods:**

This randomised, double-blind, placebo-controlled, two-way crossover study comprised two 28-day treatment periods separated by a 28-day washout. Patients (*N* = 44) inhaled CHF6523 in one period, and placebo in the other, both twice daily. The primary objective was to assess the safety and tolerability of CHF6523; the secondary objective was to assess CHF6523 pharmacokinetics. Exploratory endpoints included target engagement (the relative reduction in phosphatidylinositol (3,4,5)-trisphosphate [PIP_3_]), pharmacodynamic evaluations such as airflow obstruction, and hyperinflation, and to identify biomarker(s) of drug response using proteomics and transcriptomics.

**Results:**

CHF6523 plasma pharmacokinetics were characterised by an early maximum concentration (C_max_), reached 15 and 10 min after dosing on Days 1 and 28, respectively, followed by a rapid decline. Systemic exposure on Day 28 showed limited accumulation, with ratios < 1.6 for C_max_ and area under the curve from 0 to 12 h post-dose, and with steady state achieved on Day 20. Target engagement was confirmed by a significant 29.7% reduction from baseline in induced sputum PIP_3_ (29.5% reduction vs. placebo; adjusted ratio 0.705 [0.580, 0.856]; *p* = 0.001), but this did not translate into an anti-inflammatory pharmacodynamic effect, as assessed through measures including biomarkers and multi-omics. Additionally, although CHF6523 was generally well-tolerated, 95.2% of patients reported cough as an adverse event, most mild to moderate and resolving within one-hour post-dose.

**Conclusions:**

These data, together with those from other PI3K inhibitors, suggest that PI3Kδ is not a suitable pathway for the management of COPD, as the achieved target engagement did not translate into any pharmacodynamic anti-inflammatory effect.

**Trial registration:**

ClinicalTrials.gov (NCT04032535); posted 23rd July 2019.

**Supplementary Information:**

The online version contains supplementary material available at 10.1186/s12931-024-02999-5.

## Background

Even in the ‘stable’ state, chronic obstructive pulmonary disease (COPD) is an inflammatory condition, with systemic and airway inflammation increasing further during exacerbations [1]. Despite the availability of a range of effective therapies, patients with COPD continue to experience exacerbations. For example, in a UK retrospective database analysis of patients who initiated the triple combination of an inhaled corticosteroid, a long-acting β_2_-agonist, and a long-acting muscarinic antagonist (ICS/LABA/LAMA), 31.8% had at least one moderate or severe COPD exacerbation in the subsequent year [2]. However, the mechanisms for amplified inflammation in the stable and exacerbation states are not fully understood [1], prompting the search for anti-inflammatory treatments that target novel pathways.

Kinases, most notably the phosphoinositide 3-kinases (PI3K), are thought to be involved in chronic airway inflammation [3, 4], and this pathway is proposed as a critical regulator of inflammation and oxidative stress response in COPD [5]. There are a number of PI3K isoforms [4]. The delta isoform (PI3Kδ) is associated with recurrent respiratory infections and progressive airway damage, with a gain-of-function mutation in its catalytic subunit that activates the pathway [6]. In an allergic mouse model, PI3Kδ inhibition dampened airway inflammatory responses, including reducing the accumulation of inflammatory eosinophils [7].

Two inhaled PI3K inhibitors have previously been investigated in asthma and/or COPD – nemiralisib and AZD8154. Nemiralisib is a PI3Kδ inhibitor that demonstrated target engagement in healthy smokers [8]. However, neither the primary nor secondary endpoints were met in a Phase IIb study in patients with exacerbating COPD, and the most common adverse event was cough, experienced post-inhalation and dose-related [9]. In addition, while in one study nemiralisib demonstrated an impact on markers of neutrophil migration in patients with stable COPD, these findings were not duplicated in a subsequent study involving exacerbating COPD [10]. Furthermore, in a study conducted in activated PI3Kδ syndrome, there were no meaningful changes in target engagement or downstream inflammatory markers in induced sputum following nemiralisib treatment, with cough again the most common adverse event [11], and development was therefore halted. AZD8154 is a dual γ/δ PI3K inhibitor, which has demonstrated efficacy in a rat model of airway inflammation [12], although a Phase II study in patients with asthma was withdrawn prior to recruiting any patients due to “emerging preclinical toxicology findings” [13].

CHF6523 is an inhaled PI3Kδ inhibitor that, in a series of preclinical studies in rat or mouse models of airway inflammation, demonstrated potent inhibition of eosinophilic/type-2 inflammation comparable to that of nemiralisib. Given the positive results from these preclinical studies, CHF6523 was investigated in a three-part, first-in-human study. In the two first parts of the study, CHF6523 was administered to adult healthy volunteers in a single ascending dose design (Part 1) and then a multiple ascending dose design (Part 2). In both of these parts, CHF6523 demonstrated an acceptable safety profile, although some subjects experienced mild, inhalation-related cough that was not considered clinically significant, and were not reported as adverse events. The third part of the study, which is described in this manuscript, focused on patients with stable COPD producing sputum (i.e., chronic bronchitis phenotype), and who had evidence of type-2 inflammation.

## Methods

This was a randomised, double-blind, placebo-controlled, two-way crossover study. After completing a screening period, eligible patients were randomised equally to one of two treatment sequences, each comprising two 28-day treatment periods separated by a 28-day washout, inhaling CHF6523 in one period, and placebo in the other, both twice daily.

All assessment timings were based on the morning dose. Pre-dose on Days 1 and 28 of each treatment period, blood samples were taken for pharmacokinetic analyses, followed by forced oscillation (including slow vital capacity manoeuvres for inspiratory capacity [IC] and vital capacity [VC]), spirometry (forced expiratory volume in 1 s [FEV_1_] and forced vital capacity [FVC]), and the COPD Assessment Test (CAT). Blood samples were also taken up to 12 h post-dose on Days 1 and 28 for pharmacokinetic analyses, with blood samples for biomarker assessment pre-dose on Day 1 and 2 h post-dose on Day 28, and spirometry pre-dose on Days 20 and 24. Sputum was induced prior to each treatment period for baseline determination of biomarkers, and post-dose on Day 20 for collection of phosphatidylinositol (3,4,5)-trisphosphate (PIP_3_), and on Days 24 and 28 for collection of biomarkers. Target engagement of a PI3Kδ inhibitor is demonstrated in terms of a reduction in the relative proportion of sputum PIP_3_ in the total amount of phosphatidylinositol (4,5)-bisphosphate (PIP_2_) plus PIP_3_.

Given the importance of sputum data in the study, there was a special focus on sputum sample quality. Eligible patients were spontaneous sputum producers, i.e., they were able to produce an adequate induced sputum sample of at least 300 mg with a cell viability of at least 70% (with less than 30% epithelial cells) at screening. In addition, sputum quality was evaluated centrally at the sputum laboratory of the Pulmonary Research Institute, Grosshansdorf, Germany. See the supplement for additional details.

Safety and tolerability were evaluated throughout the study in terms of the occurrence of adverse events, blood chemistry, haematology and urinalysis, vital signs, and 12-lead electrocardiograms. Because of the occurrence of cough in healthy volunteers in Parts 1 and 2, cough episodes were monitored by asking patients to grade episodes on a visual analogue scale (VAS), and to report any episodes in a daily diary. Throughout the study, patients were permitted to use salbutamol as rescue medication, but not within 6 h prior to any spirometry assessment – any rescue medication intake was also captured in the daily diary.

Pharmacokinetic assessments were performed under fasting conditions. On days when pharmacokinetic samples were taken, study drug was administered in the morning at the clinical site after a fast of ≥ 10 h (water was allowed), with food or fluid intake not allowed until 1 h post-dose, with the exception of 100 mL of water if the patient coughed as a result of inhalation. Starting from 1 h post-dose and for the following 6 h, patients drank ≥ 240 mL of water every 2 h, with breakfast and lunch served approximately 3 and 5 h after morning study drug, and, on Day 1, dinner at least 2 h before or 1 h after evening drug administration. No intake of alcohol, grapefruit, or xanthine/caffeine containing beverages or food was allowed from 48 h prior to each intake of study medication and until the last visit procedure, with strenuous exercise prohibited for 24 h before any spirometry measurement.

The study was approved by the independent ethics committees at each institution (listed in the supplement), was performed in accordance with the Declaration of Helsinki and Good Clinical Practice, and was registered at ClinicalTrials.gov (NCT04032535; 23rd July 2019). The protocol was amended three times after recruitment started, only one of which involved any substantial changes – permitting vaccination during the washout period between the two treatment periods, and specifying that IC and VC data were recorded from forced oscillation.

### Participants

In addition to a requirement to be spontaneous sputum producers, eligible patients were diagnosed with COPD at least 12 months prior to inclusion, were current or ex-smokers with a smoking history of ≥ 10 pack-years, blood eosinophil count ≥ 150 cells/µL, post-bronchodilator FEV_1_ 30–70% predicted and FEV_1_/FVC < 0.70, were symptomatic (CAT total score ≥ 10), and had been receiving inhaled triple therapy (ICS/LABA/LAMA) for ≥ 6 months prior to entry. The main exclusion criteria were a current diagnosis of asthma, a COPD exacerbation in the 6 weeks prior to entry, and the use of any other COPD maintenance therapy other than ICS/LABA/LAMA in the 6 months prior to entry. The full list of inclusion and exclusion criteria is in the supplement. All patients provided written informed consent prior to any study-related procedure.

### Interventions

The treatments administered were CHF6523 2 mg twice daily or matching placebo, both via capsule-based dry-powder inhaler, with all patients continuing to take the ICS/LABA/LAMA that they were receiving on entry to the study as background COPD therapy for the duration of follow-up. The CHF6523 dose was selected on the basis of pharmacokinetic and pharmacodynamic modelling from healthy volunteer data in Parts 1 and 2 (it was slightly above the predicted therapeutic dose, which was a total daily dose of 3 mg). Patients were randomised according to a balanced block scheme according to a pre-established randomisation list prepared by the sponsor. Patients, investigators and their staff, monitors and the sponsor’s clinical team were all blinded to treatment.

### Outcomes

The primary objective was to assess the safety and tolerability of CHF6523 in patients with COPD. The secondary objective was to investigate the pharmacokinetic profile of CHF6523 in plasma in terms of:

- On Day 1: Area under the curve from 0 to 30 min and 0–12 h post-dose (AUC_0–30 min_ and AUC_0–12 h_), maximum concentration (C_max_), and time to maximum concentration (t_max_).
- On Days 20 and 24: pre-dose concentration (C_trough_).
- On Day 28: C_trough_, AUC_0–30 min_, AUC_0–12 h_, C_max_, minimum concentration (C_min_), t_max_, time to minimum concentration (t_min_), average concentration (C_av_), accumulation ratios (R_ac_) for C_max_ and AUC_0–12 h_, and the apparent total body clearance (calculated as dose/area under the curve from time 0 extrapolated to 12 h post-dose) at steady state (CL/F_ss_).

Exploratory endpoints included PIP_3_, cell counts and target biomarkers of inflammation in induced sputum, plasma biomarkers, airflow obstruction, hyperinflation and lung mechanics, symptoms, and to identify biomarker(s) of drug response using proteomics (Olink and mass-spectrometry) and transcriptomics (RNA-Seq). In addition, the number and severity of cough episodes, and the use of rescue medication (percentage of days with no use and average daily use) were evaluated as exploratory endpoints.

### Sample size and statistical methods

The study was not formally powered, but a total of 42 evaluable patients was deemed adequate to assess the safety and tolerability of CHF6523. Assuming a drop-out of 30%, 60 patients were to be randomised.

Fold-change from baseline in cell counts, target biomarkers in sputum and blood, and PIP_3_ were log-transformed and analysed using an analysis of covariance (ANCOVA) model, with treatment, patient, and period as fixed effects, and baseline value as covariate. Changes from baseline in spirometry, forced oscillation and CAT endpoints were analysed using a similar ANCOVA model, but without log transformation. Rescue medication data were analysed using an analysis of variance model, including treatment, period and patient as fixed effects. Other data, including the proportion of days on which patients coughed and the cough VAS results, are presented descriptively only. Further details, including the transcriptomics, mass-spectrometry and Olink proteomics, bioinformatics and multi-omic data integration are in the supplement.

The safety set, used in the analysis of all safety variables, was all randomised patients who received at least one dose of study drug. The pharmacokinetic and pharmacodynamic sets were all patients in the safety set excluding those without any valid pharmacokinetic or pharmacodynamic measurements, respectively, or who had major protocol deviations that impacted the pharmacokinetic or pharmacodynamic assessments, respectively. Pharmacokinetic variables were analysed using the pharmacokinetic set; exploratory pharmacodynamic variables were analysed in the pharmacodynamic set. Given the cross-over design, the inclusion of patients in the analysis sets was defined on a per-period basis.

## Results

The study ran between 4 November 2021 and 5 December 2022, at five specialist investigative centres in Germany. Of 63 patients screened, 44 were randomised, 33 of whom (75.0%) completed the study (nine withdrew due to adverse events with onset after treatment commenced, and one withdrew consent; a further patient withdrew due to an adverse event that started during the run-in period). Although it was planned to randomise 60 patients, recruitment into the study was terminated, partly due to the coronavirus disease 2019 (COVID-19) pandemic and partly due to investigator concerns over the occurrence of cough adverse events (see the ‘safety’ results section). Baseline characteristics of the randomised patients are shown in Table 1.

**Table 1:** Baseline characteristics (safety set)

Parameter	All patients (*N* = 44)
Age, years	67.0 (7.6)
Sex, female	13 (29.5%)
Race, white	44 (100%)
Body mass index, kg/m^2^	27.2 (4.1)
Smoking status	
Current smoker	17 (38.6%)
Ex-smoker	27 (61.4%)
Time since first COPD diagnosis, years	13.3 (5.6)
Number of COPD exacerbations in the previous 12 months	0.4 (0.6)
CAT total score	21.4 (6.3)
Blood eosinophils, cells/µL	303.2 (327.2)
Post-bronchodilator FEV_1_	
Absolute, L	1.496 (0.417)
Percent predicted	50.3 (11.8)
Post-bronchodilator FEV_1_/FVC, %	47.4 (10.9)

Data are mean (standard deviation) or number (percent). COPD, chronic obstructive pulmonary disease; CAT, COPD Assessment Test; FEV_1_, forced expiratory volume in 1 s; FVC, forced vital capacity

### Pharmacokinetics

**Fig. 1:**
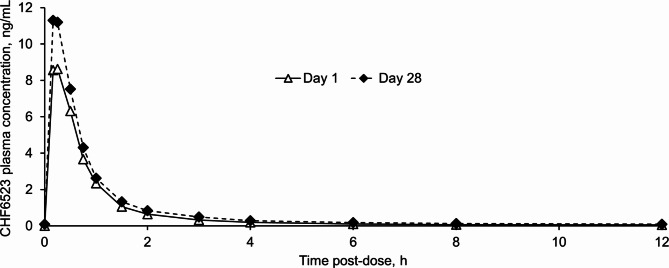
CHF6523 plasma concentration–time profile (pharmacokinetic set). Data are mean, and were available for 40–41 patients on Day 1, and for 28–29 patients on Day 28

The mean CHF6523 plasma concentration increased rapidly following dosing on both Day 1 and Day 28, reaching a peak at 15 min on Day 1, and 10 min (the first post-dose timepoint) on Day 28 (Fig. 1). Consistent with the mean plasma concentration–time profile, median t_max_ values were 0.25 and 0.18 h (15 and 11 min) on Days 1 and 28 (Table 2). Plasma C_max_ and AUC values were higher on Day 28 than after a single dose (Day 1), with mean C_max_ and AUC_0 − 12 h_ R_ac_ values of 1.47 and 1.52, respectively. Plasma steady state was evident from Day 20.

**Table 2:** CHF6523 plasma pharmacokinetics (pharmacokinetic set)

Parameter	All patients*
AUC_0–30 min_, ng.h/mL	
Day 1	3.28 (1.64)
Day 28	4.21 (2.05)
AUC_0–12 h_, ng.h/mL	
Day 1	8.10 (4.11)
Day 28	10.50 (5.32)
R_ac_	1.520 (0.826)
C_max_, ng/mL	
Day 1	9.10 (4.42)
Day 28	11.70 (5.54)
R_ac_	1.470 (0.775)
t_max_, h	
Day 1	0.25 (0.15–2.00)
Day 28	0.18 (0.15–0.27)
C_trough_, ng/mL	
Day 20	0.10 (0.05)
Day 24	0.09 (0.06)
Day 28	0.09 (0.06)
Day 28	
C_min_, ng/mL	0.09 (0.03)
t_min_, h	11.92 (8.13–12.17)
C_av_, ng/mL	0.88 (0.45)
CL/F_ss_, L/h	239 (119)

Data are mean (standard deviation) except t_max_ and t_min_, which are median (range). *Data available from 40–41 patients on Day 1, 31 on Day 20, 29 on Day 24, and 28–29 on Day 28. AUC_0–30 min_ and AUC_0–12 h,_ area under the curve from time 0–30 min and 0–12 h post-dose; R_ac_, accumulation ratio; C_max_, maximum concentration; t_max_, time to maximum concentration; C_trough_, pre–dose concentration; C_min_, minimum concentration; t_min_, time to minimum concentration; C_av_, average concentration; CL/F_ss_, the apparent total body clearance (calculated as dose/area under the curve from time 0 extrapolated to 12 h post-dose) at steady state

### PIP_3_

Overall sputum quality collected in this study was high, with mean viability in adequate samples of 93%, and 5% squamous cells. In the CHF6523 group, there was a 29.7% decrease from baseline in sputum PIP_3_ levels at Day 20 (adjusted geometric mean fold change 0.703 [95% CI 0.606, 0.814]), compared with no change in the placebo group (0.3%; 0.997 [0.883, 1.126]), with a resulting significant 29.5% difference between groups (adjusted ratio 0.705 [0.580, 0.856]; *p* = 0.001).

### Induced sputum and plasma biomarkers

**Fig. 2:**
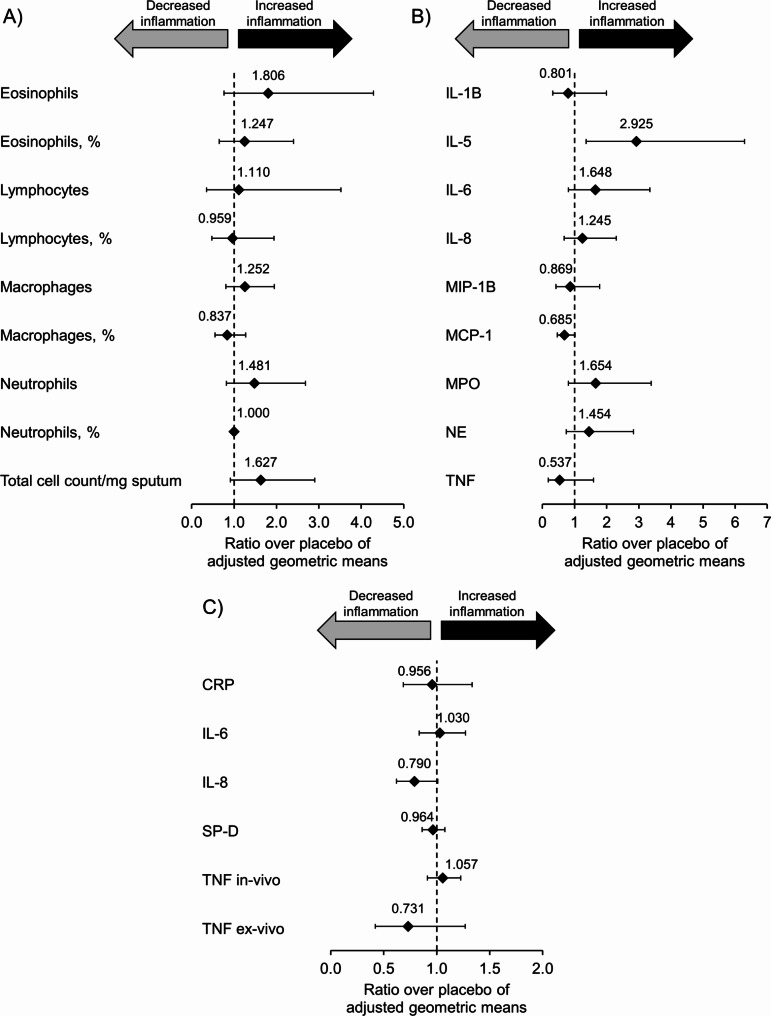
(**A**) Cell count and (**B**) target biomarkers in sputum, and (**C**) target biomarkers in plasma (pharmacodynamic set). Sputum cell count data available from 30 and 34 patients with CHF6523 and placebo, respectively, target biomarker sputum data available from 18–31 and 25–34 patients, and target biomarker plasma data available from 28–30 and 34–36 patients. IL, interleukin; MIP-1B, macrophage inflammatory protein 1B; MCP-1, monocyte chemoattractant protein-1; MPO, myeloperoxidase; NE, neutrophil elastase; TNF, tumour necrosis factor; CRP, C-reactive protein; SP-D, surfactant protein D

There were no statistically significant (*p* < 0.05) CHF6523–placebo differences in sputum cell counts or target biomarkers in sputum, with the exception of interleukin 5 (IL-5), levels of which were significantly higher with CHF6523 – i.e., increased inflammation, rather than the expected anti-inflammatory effect (Fig. 2A and B). Similarly, there were no statistically significant (*p* < 0.05) CHF6523–placebo differences in the target biomarkers in plasma (Fig. 2C).

### Airflow obstruction, hyperinflation, lung mechanics, and symptoms

**Fig. 3:**
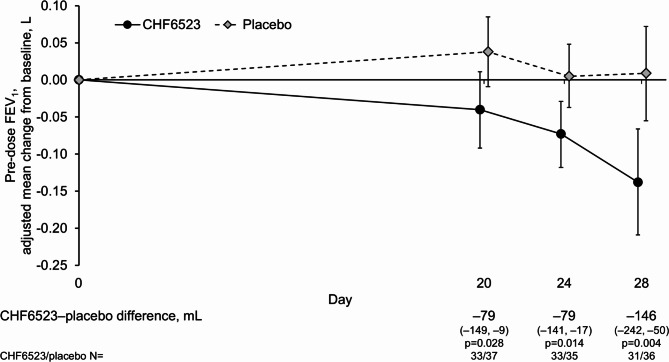
Change from baseline in pre-dose FEV_1_ (pharmacodynamic set). Data are adjusted mean and 95% confidence intervals. FEV_1_, forced expiratory volume in 1 s

**Fig. 4:**
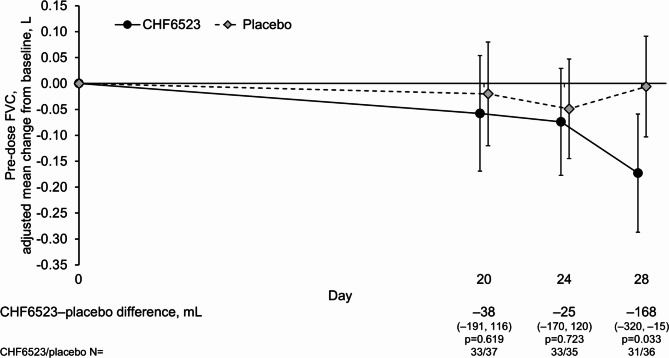
Change from baseline in pre-dose FVC (pharmacodynamic set). Data are adjusted mean and 95% confidence intervals. FVC, forced vital capacity

Mean pre-dose FEV_1_ and FVC decreased from baseline over the follow-up period during treatment with CHF6523, but was maintained with placebo, with statistically significant CHF6523–placebo differences in FEV_1_ at all three visits ranging from − 79 to − 146 mL, and in FVC of − 168 mL on Day 28 (Figs. 3 and 4). However, there were no marked differences in the slow vital capacity assessments, and the only statistically significant CHF6523–placebo difference in the oscillometry parameters was inspiratory flow (29 mL/s; *p* = 0.015; Table S1). Furthermore, CAT total score did not change from baseline with either treatment, and rescue medication use was low, with no differences between groups (Table S1).

### Proteomics

#### Olink protein biomarker platform in sputum

**Fig. 5:**
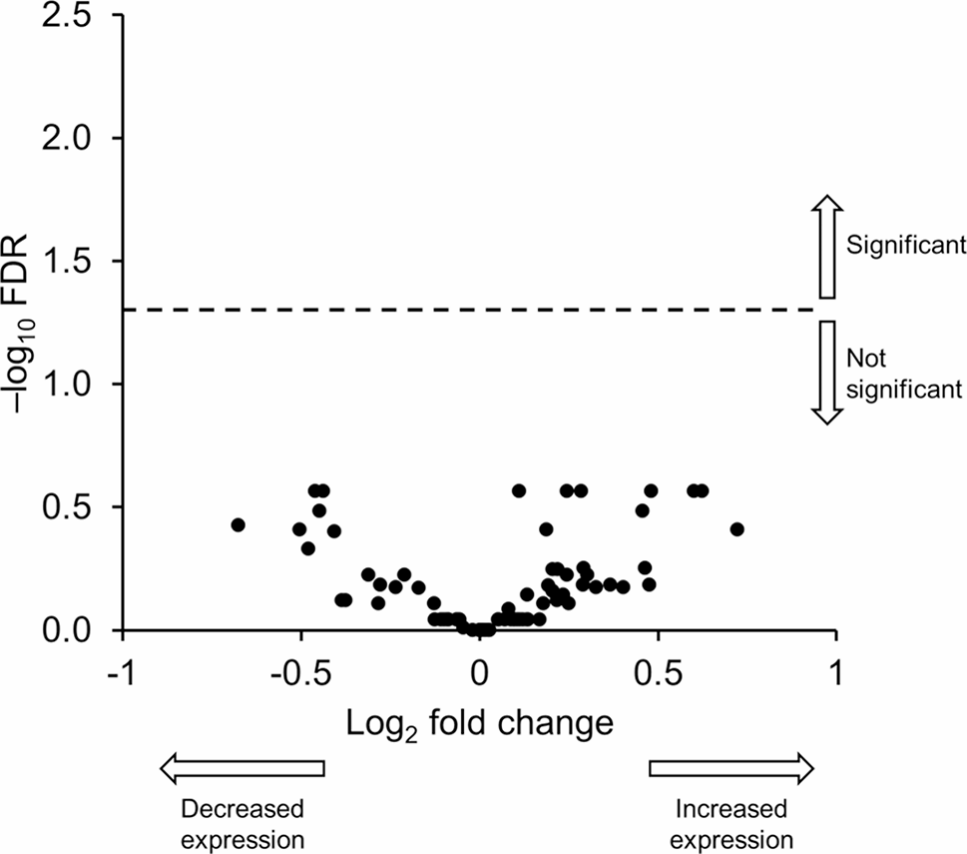
Olink proteomics: Modulation of 74 proteins in sputum following administration of CHF6523 in the two Olink panels (pharmacodynamic set). FDR, false discovery rate

Data from 27 patients were included in the Target 96 Inflammation panel and from 28 patients in the Target 48 Cytokine panel. Of the 74 proteins evaluated, none were significantly modulated by treatment (false discovery rate [FDR] < 0.05; Fig. 5). Using these two panels, it was possible to validate five biomarkers in sputum, confirming the sputum target biomarker data showing similar fold changes to those reported in Fig. 2B (0.771, 1.516, 1.151, 0.732, and 0.821 in the Olink panels for IL-1B, IL-6, IL-8, monocyte chemotactic protein 1 [MCP-1], and tumour necrosis factor [TNF], respectively).

#### Tandem mass tag mass spectrometry proteomics in sputum

**Fig. 6:**
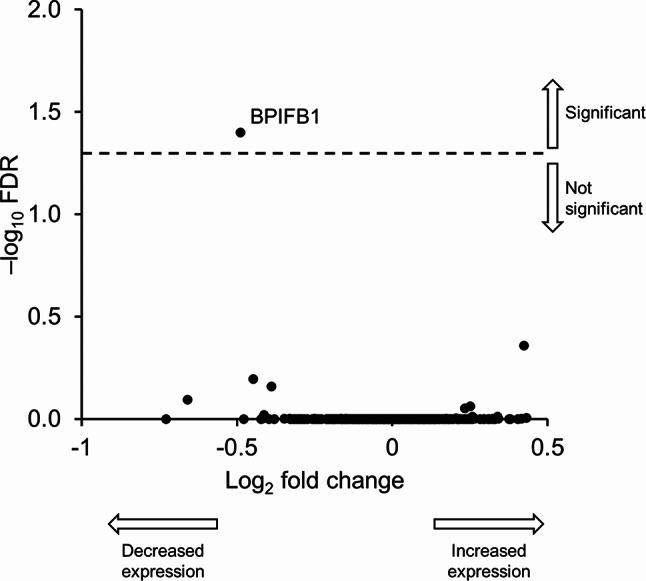
Untargeted proteomics: Modulation of proteins in sputum following administration of CHF6523 (pharmacodynamic set). FDR, false discovery rate; BPIFB1, BPI fold-containing family B member 1

An untargeted mass spectrometry with tandem mass tag (TMT) labelling was used to investigate differentially expressed proteins (DEPs) from 20 patients in induced sputum. Of the 413 proteins evaluated (post-quality control), only BPI fold-containing family B member 1 (BPIFB1) was significantly modulated by treatment (Fig. 6; FDR < 0.05, mixed effect modelling).

### Transcriptomics

**Fig. 7:**
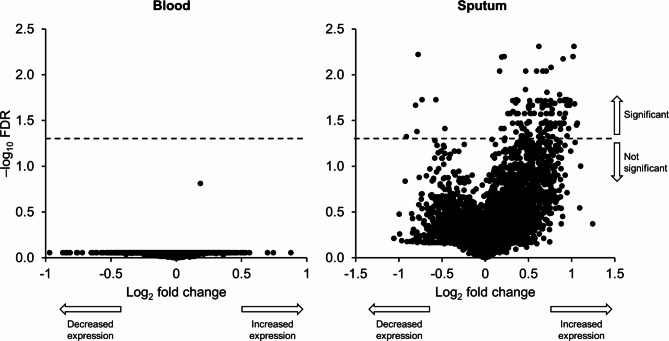
Transcriptomics: Differential expression of genes in blood and sputum following administration of CHF6523 (pharmacodynamic set). FDR, false discovery rate

More than 38,000 genes were evaluated in blood from 24 patients, and in sputum from 23 patients using RNA-seq technology. No differentially expressed genes were observed in the blood samples (Fig. 7). In sputum 126 genes were identified as significantly differentially expressed by the fitted model (FDR < 0.05) following administration of CHF6523, 119 of which were upregulated (Fig. 7); 66 were non-protein coding. However, only six genes had |log_2_FC| >1 (range 1.019–1.067), meaning that the majority of genes had low changes in expression. From subsequent functional enrichment, the gene ontology biological processes and molecular functions showed that the genes that were differentially expressed in sputum were primarily involved in Toll-like receptor (TLR) 2 binding and lipopeptide binding (Table S2), with three TLR genes available in the gene list (TLR6: log2FC 0.52, FDR = 0.0451; TLR10: 0.48, FDR = 0.0418; TLR1: 0.71, FDR = 0.0155). However, the change of expression of all three genes was low (|log_2_FC| <1 and FDR < 0.05).

### Multi-omic integration

**Fig. 8:**
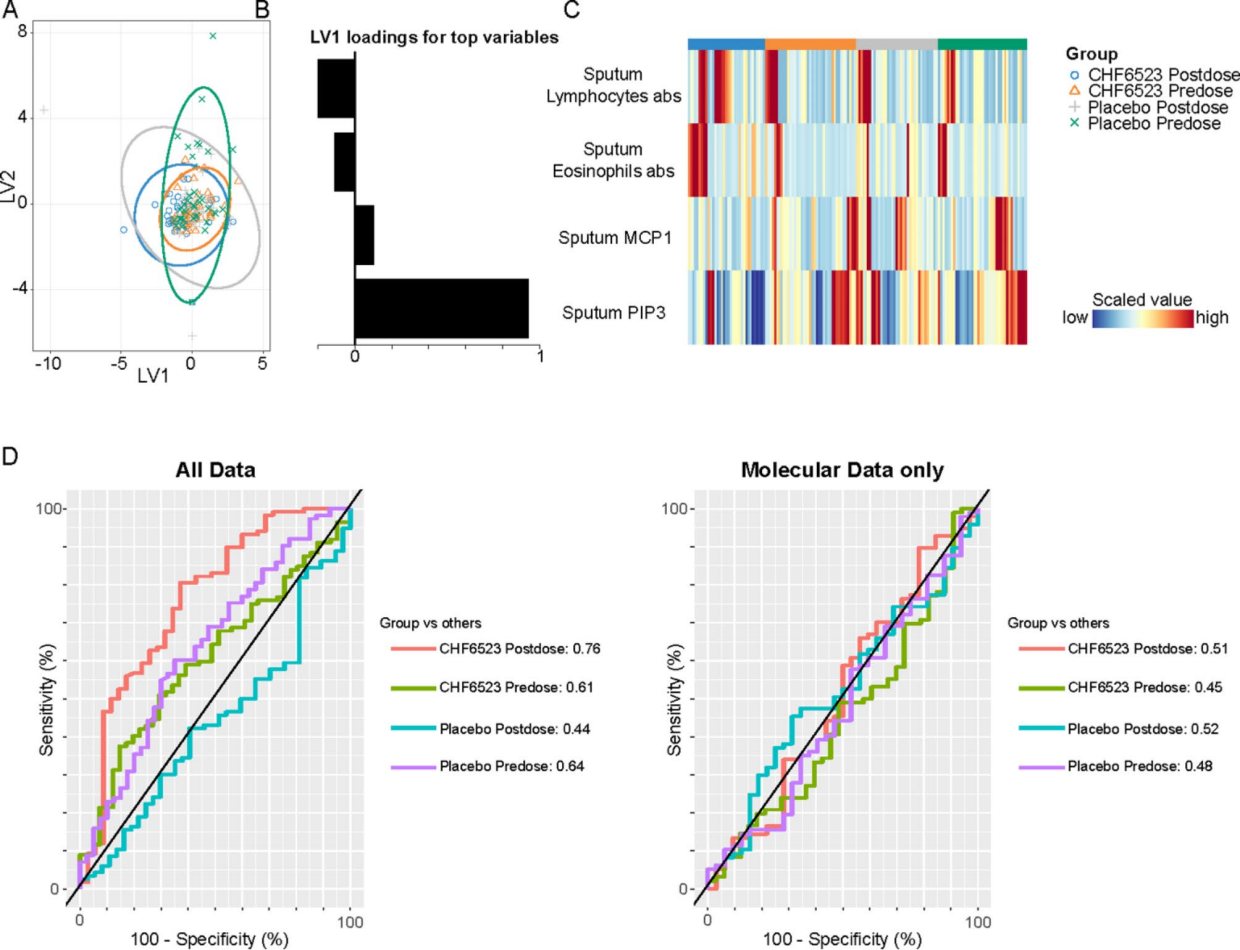
Multi-omic integration profiles. (A) Projection of the top two latent variables (LV). (B) Top loadings on LV1. (C) Heatmap of the top variables in LV1 in all samples. (D) Area under the receiving operating curve of each group (vs. the three others), when including all data (left) and when considering only molecular data (right). PIP_3_, phosphatidylinositol (3,4,5)-trisphosphate; PIP_2_, phosphatidylinositol (4,5)-bisphosphate; MCP1, monocyte chemotactic protein 1

Given there was no marked treatment effect for the individual level transcriptomics and proteomics data, a multi-omic integrated analysis was performed using a machine learning evaluation of patient data integrating sputum Olink panels, mass spectrometry sputum proteomics, blood and sputum RNA-seq, plasma and sputum target biomarkers, PIP_3_, sputum and blood cell counts, and blood chemistry. A projection of all samples based on best-fit models indicated substantial overlap between baseline and post-dose for placebo and CHF6523 (Fig. 8A).

Based on the best performing models, and stratifying the four groups (CHF6523 baseline and post-dose, and placebo baseline and post-dose) there was a medium classification power for CHF6523 post-dose vs. the three other groups, with sputum PIP_3_ emerging as the primary predictive factor (area under the receiving operating curve [AUROC] of each group vs. the three others: CHF6523 post-dose 0.76; CHF6523 baseline 0.61; placebo post-dose 0.44; placebo baseline 0.64; Fig. 8B–D). When only proteomics and transcriptomics data were considered, there was an even more apparent overlap between groups, and the ability of the model to effectively classify samples was very poor (AUROC 0.45–0.52; Fig. 8D). Collectively, these observations suggest that molecular features, even when integrated, had limited treatment-induced predictive power, consistent with the findings from the individual omics-based analyses. Nonetheless, sputum PIP3 maintained a significant predictive value as the primary variable influenced by the treatment. Individual comparison of baseline vs. post-dose for each treatment (CHF6523 or placebo) demonstrated qualitatively similar results (Figure S1).

### Safety

All but one patient reported adverse events when receiving CHF6523, most commonly cough and an abnormal product taste (Table 3). The majority of adverse events were mild or moderate in severity, and were not considered serious, with none resulting in death. All of the cough and taste adverse events that occurred with CHF6523 were considered related to treatment, with five patients withdrawing from the study due to cough. There were no clinically relevant CHF6523–placebo differences in any of the other safety evaluations.

Patients coughed on an adjusted mean 74.0% (95% CI 63.5%, 84.5%) days when receiving CHF6523, compared with 4.8% (–5.4%, 15.0%) days with placebo. The majority of cough episodes occurred during or immediately after inhalation, with only 3.2% of cough events not resolving within 1 h after inhalation. The mean VAS scores with CHF6523 were 30.3 (95% CI 21.3, 39.3) and 31.3 (22.1, 40.4) mm in the morning and evening, compared with 0.7 (–0.2, 1.6) and 0.6 (–0.1, 1.3) mm with placebo (the VAS was 100 mm in length, with 0 being ‘no cough’, and 100 being ‘worst cough ever’).

**Table 3:** Adverse events, overall and most common (occurring in > 2 patients with either treatment; safety set)

Patients (%)	CHF6523 (*N* = 42)	Placebo (*N* = 40)
Adverse events	41 (97.6%)	16 (40.0%)
Cough	40 (95.2%)	1 (2.5%)
Abnormal product taste	14 (33.3%)	0
Dyspnoea	9 (21.4%)	1 (2.5%)
Chest discomfort	9 (21.4%)	0
COPD exacerbation	4 (9.5%)	4 (10.0%)
Increased sputum	5 (11.9%)	1 (2.5%)
COVID-19	5 (11.9%)	0
Headache	5 (11.9%)	0
Treatment-related adverse events	40 (95.2%)	1 (2.5%)
Cough	40 (95.2%)	0
Product taste abnormal	14 (33.3%)	0
Chest discomfort	9 (21.4%)	0
Dyspnoea	9 (21.4%)	0
Increased sputum	5 (11.9%)	1 (2.5%)
Headache	3 (7.1%)	0
Severe adverse events	11 (26.2%)	2 (5.0%)
Cough	7 (16.7%)	0
Serious adverse events	2 (4.8%)	2 (5.0%)
Serious treatment-related adverse events	0	0
Adverse events leading to study discontinuation	6 (14.3%)	3 (7.5%)
Cough	5 (11.9%)	0
Adverse events leading to death	0	0

COVID-19, coronavirus disease 2019

## Discussion

The primary objective of the study was to assess the safety and tolerability of CHF6253 in patients with COPD. Although there was a notable imbalance in the occurrence of adverse events, most events with both treatments were mild or moderate in severity, and were not considered serious, and there were no safety signals in terms of laboratory or cardiac parameters. For the secondary objective, to investigate the pharmacokinetic profile of CHF6523 in plasma, plasma steady state was reached by Day 20, with limited systemic accumulation.

All but two of the patients had a treatment-related cough adverse event during the CHF6523 treatment period. The majority of these events were related to study drug inhalation and resolved within 1 h post-dose. Although the events tended to be mild or moderate in severity and relatively short-lasting, this adverse event was the most common reason for patients to withdraw from the study. Further preclinical investigation into cough was undertaken subsequent to this study. In an electrophysiology in-vitro examination, CHF6523 had no discernible impact on the various ion channels implicated in the cough reflex. However, it did trigger coughing in an in-vivo guinea pig model. Changing the formulation to use alternative salts rendered CHF6523 ineffective in influencing cough, indicating that the coughing phenomenon may be attributable to the compound’s specific salt form, rather than being an intrinsic characteristic of the molecule or mechanism of action.

Even though the various sputum evaluations were only exploratory endpoints, investigators devoted substantial effort to sputum collection and processing. This resulted in the samples being of high quality, as shown by the mean viability of 93%, and reinforces the confidence in the results obtained. The induced sputum PIP_3_ data, with administration of CHF6523 resulting in a 29.7% decrease from baseline (and a 29.5% reduction vs. placebo) at Day 20, clearly demonstrate successful target engagement. Nevertheless, this engagement did not translate into a pharmacodynamic effect in terms of reduced inflammation, with very limited statistically significant differences of molecular data between CHF6523 and placebo, indicating a lack of meaningful biological response to the drug, likely explaining the absence of a positive effect.

The impact of CHF6523 on PIP_3_ mirrors that of nemiralisib in a healthy volunteer study, with a 35.7% reduction at Day 12 [14]. Likewise, consistent with our study, nemiralisib did not significantly reduce sputum IL-6 and IL-8 levels compared to placebo in patients with COPD [15]. Similarly, in a study involving patients with asthma, the administration of nemiralisib did not yield statistically significant reductions in sputum IL-5, IL-13, IL-6 or IL-8 compared to placebo [16]. As a result, when reviewing the existing literature, we can reasonably infer some parallels between nemiralisib and CHF6523 in terms of their lack of anti-inflammatory pharmacodynamic effects despite successful target engagement and positive data observed in preclinical models of airway inflammation – and both molecules were associated with post-inhalation cough. The nemiralisib COPD studies recruited patients who either received any concomitant COPD therapy (including mono-bronchodilators) [15], or had experienced an acute COPD exacerbation [9], and neither study demonstrated an effect of nemiralisib on the clinical efficacy parameters assessed. As this lack of an effect was potentially due to the populations recruited, the current study specifically recruited patients who had been receiving triple ICS/LABA/LAMA therapy for ≥ 6 months, and who had evidence of type-2 inflammation (blood eosinophils ≥ 150 cells/µL).

However, CHF6523 demonstrated negligible impact in the proteomics assay, with the exception of a reduction in BPIFB1, a protein typically elevated in patients with COPD [17]. Additionally, only 126 out of over 38,000 genes evaluated in sputum showed a differential expression, predominantly with low levels of variation. Furthermore, in the multi-omics analysis the primary factor accounting for the variability induced by CHF6523 was PIP_3_, the drug’s molecular target, corroborating the previous individual-level findings in a comprehensive, unbiased, and untargeted manner, with other molecular features remaining mostly unaffected. In addition, CHF6523 didn’t impact CAT total score, rescue medication use, or the oscillometry endpoints. Notably, there was a deterioration in FEV_1_ during CHF6523 treatment, and an elevation in the type-2 mediator IL-5 levels in sputum.

The perplexing lack of any discernible anti-inflammatory effect despite PI3K target engagement, either biomarkers or clinical efficacy endpoints, poses a challenge, given the preclinical evidence suggesting the potential alleviation of inflammatory and oxidative stress conditions by targeting this pathway [5, 7]. This absence of an anti-inflammatory pharmacodynamic response may be linked to the mechanism of action of PI3K inhibitors, potentially attributable to the redundancy of pathways wherein inhibiting PI3K could trigger the activation of alternative pathways in COPD. Additionally, the divergent or opposing effects of PI3K inhibition across different cell types could contribute to the overall minimal impact on observed anti-inflammatory pharmacodynamic parameters. The alternative hypothesis of cough counteracting the anti-inflammatory effect or even reversing some features (as FEV_1_ and sputum IL-5) seems less probable, particularly as acute cough episodes were mostly mild to moderate and resolved within 1 h post inhalation. Overall, the inability to elicit an anti-inflammatory pharmacodynamic effect despite confirmed target engagement corroborates findings from previous studies with other PI3K inhibitors, suggesting that this pharmacological pathway is unlikely to be an effective target for respiratory inflammatory conditions.

The main limitation of the study is that only 44 patients were recruited, 33 of whom completed both periods. Although not formally powered, this is less than the estimated sample size of 60 recruited patients, with 42 completing. However, given the clear demonstration of target engagement, if the study had recruited a larger number of patients, it is still unlikely that any treatment effect would have been demonstrated on the exploratory pharmacodynamic endpoints, given the detailed profiling at both local (sputum) and systemic (blood) levels. In addition, the population recruited was relatively homogeneous, with all recruited patients being white, and most male, ex-smokers.

## Conclusion

Overall, therefore, these data, together with those for other PI3K inhibitors, suggest that PI3Kδ is unlikely to be a target for the management of COPD. Even if a reformulation of CHF6523 could reduce the impact of the cough adverse event, and despite the otherwise good overall safety profile, the target engagement demonstrated in this study did not translate into any pharmacodynamic effect in terms of a reduction in inflammation. These findings underscore the importance of adopting a systematic, data-driven translational approach to evaluating a molecule’s potential early in the clinical development phase.

## Electronic supplementary material

Below is the link to the electronic supplementary material.

Supplementary Material 1

## Data Availability

Chiesi commits to sharing with qualified scientific and medical researchers, conducting legitimate research, the anonymised patient-level and study-level data, the clinical protocol and the full clinical study report of Chiesi Farmaceutici SpA-sponsored interventional clinical trials in patients for medicines and indications approved by the European Medicines Agency and/or the US Food and Drug Administration after 1st January 2015, following the approval of any received research proposal and the signature of a Data Sharing Agreement. Chiesi provides access to clinical trial information consistently with the principle of safeguarding commercially confidential information and patient privacy. Other information on Chiesi’s data sharing commitment, access and research request’s approval process are available in the Clinical Trial Transparency section of http://www.chiesi.com/en/research-and-development/.

## Abbreviations

ANCOVA: Analysis of covariance
AUC_0–12h_: Area under the curve from time 0–12 h post-dose
AUC_0–30min_: Area under the curve from time 0–30 min post-dose
AUROC: Area under the receiving operating curve
BPIFB1: BPI fold-containing family B member 1
CAT: COPD Assessment Test
C_av_: Average concentration
CL/F_ss_: Apparent total body clearance
C_max_: Maximum concentration
C_min_: Minimum concentration
COPD: Chronic obstructive pulmonary disease
COVID-19: Coronavirus disease 2019
CRP: C-reactive protein
C_trough_: Pre-dose concentration
DEPs: Differentially expressed proteins
EFL: Expiratory flow limitation
FDR: False discovery rate
FEV_1_: Forced expiratory volume in 1 s
FVC: Forced vital capacity
IC: Inspiratory capacity
ICS/LABA/LAMA: Inhaled corticosteroid/long-acting β_2_-agonist/long-acting muscarinic antagonist
IL: Interleukin
MCP-1: Monocyte chemoattractant protein-1
MIP-1B: Macrophage inflammatory protein 1B
MPO: Myeloperoxidase
NE: Neutrophil elastase
PI3K: Phosphoinositide 3-kinases
PIP_2_: Phosphatidylinositol (4,5)-bisphosphate
PIP_3_: Phosphatidylinositol (3,4,5)-trisphosphate
R_ac_: Accumulation ratio
Rrs: Resistance
SP-D: Surfactant protein D
SVC: Slow vital capacity manoeuvre
TLR: Toll-like receptor
t_max_: Time to maximum concentration
t_min_: Time to minimum concentration
TMT: Tandem mass tag
TNF: Tumour necrosis factor
VAS: Visual analogue scale
VC: Vital capacity
Xrs: Reactance
Zrs: Mechanical impedance
