# Supplementary material for: CHF6523 data suggest that the phosphoinositide 3-kinase delta isoform is not a suitable target for the management of COPD
Source: Respir Res. 2024 Oct 19;25:380. doi: 10.1186/s12931-024-02999-5 (PMC11491004; doi:10.1186/s12931-024-02999-5)
Supplement: Supplementary file 1 — Supplementary Material 1 [file 12931_2024_2999_MOESM1_ESM.docx]

# CHF6523 data suggest that the phosphoinositide 3-kinase delta isoform is not a suitable target for the management of COPD

Mirco Govoni, Michele Bassi, Luca Girardello, Germano Lucci, François Rony, Rémi Charretier, Dmitry Galkin, Maria Laura Faietti, Barbara Pioselli, Gloria Modafferi, Rui Benfeitas, Martina Bonatti, Daniela Miglietta, Jonathan Clark, Frauke Pedersen, Anne-Marie Kirsten, Kai-Michael Beeh, Oliver Kornmann, Stephanie Korn, Andrea Ludwig-Sengpiel, Henrik Watz

# Additional file 1

# Ethics committees

| **Site** | **Address** |
| --- | --- |
| 2 | Ethikkonunission bel der Arztekanuner Schleswig-Holstein (AKSH)  Ethics Committee at the Medical Association of Schleswig Holstein,  Bad Segeberg, Germany |
| 3 | Landesarztekammer Hessen Ethikkonunission  Frankfurt am Main, Germany |
| 4 | Ethikkonunission bei der Arztekanuner Schleswig-Holstein (AKSH)  Ethics Committee at the Medical Association of Schleswig Holstein,  Bad Segeberg, Germany |
| 5 | Landesarztekammer Hessen Ethikkonunission  Frankfurt am Main, Germany |
| 6 | Landesarztekammer Rheinland-Pfalz Ethikkonunission  Mainz, Germany |

# Methods

## Blood collection and processing for CHF6523 pharmacokinetic analyses

Blood samples of 2 mL were collected in K_2_-EDTA tubes at pre-dose and at 10, 15, 30, and 45 min and 1, 1½, 2, 3, 4, 6, 8 and 12 h post-dose on Days 1 and 28, and pre-dose on Days 20 and 24. Collected samples were centrifuged within 1 h after blood in a refrigerated centrifuge (at about +4°C) at about 2000 x g for 10 min. The plasma obtained was transferred into two polypropylene tubes (at least 500 μL into the first, and the remaining volume into the second), then stored at or below –80°C, at the clinical centre. The tubes were then transferred (frozen) to a central laboratory, and stored at –80°C until submitted for analysis with a validated liquid chromatography with tandem mass spectrometry (LC/MS-MS) method. Two validated methods were used to cover the range of possible concentrations. The first one, the so-called low-range method, measured CHF6523 over the calibration range 0.02–1.00 ng/mL. The second, the high-range method, measured CHF6523 over the calibration range 1.00–50.0 ng/mL. Overall, the lower limit of quantification in this study (LLoQ) was of 0.02 ng/mL.

## Forced oscillation

The Resmon PRO FULL device (MGC Diagnostics/Medisoft, St Paul, MN, USA) was used to evaluate the forced oscillometry endpoints. Patients in a sitting position and with their nose clipped supported their cheeks to reduce upper airway shunting while small amplitude pressure stimuli were applied during tidal breathing, with each manoeuvre performed in triplicate. The response of the respiratory system to this stimulation was used to derive respiratory mechanical impedance (Zrs), which comprises resistance (Rrs) and reactance (Xrs). In addition, the small amplitude pressure stimuli was also applied during execution of an expiratory slow vital capacity manoeuvre (SVC) to record inspiratory capacity (IC) and vital capacity (VC).

Central and peripheral airway mechanics were measured using a multi frequency stimulating waveform at 5, 11, and 19 Hz, to derive the following:

- Rrs5 (total, inspiratory, expiratory), representing total, inspiratory and expiratory upper and lower airway resistance;
- Rrs19 (total, inspiratory, expiratory), representing lower airway resistance;
- Rrs 5 minus Rrs19 (total, inspiratory, expiratory), representing small airway resistance;
- Xrs 5 (total, inspiratory, expiratory) and Xrs 19 (total, inspiratory, expiratory), reflecting lung compliance and ventilation inhomogeneities;
- expiratory flow limitation index (ΔXrs inspiratory–expiratory), which is a measure of expiratory flow limitation (EFL), and is also expressed as the percentage of breaths where EFL was present (FL%);
- resonance frequency, i.e. the frequency at which reactance is 0.

The respiratory pattern, in terms of respiratory rate, and tidal volume were also reported.

## Blood collection and processing for biomarker evaluation

Blood samples for biomarkers assessment were collected, as follows:

- Two samples on Day 1 at pre-dose and two on Day 28 at 2 h post-dose for plasma target biomarkers;
- Two samples on Day 1 at pre-dose and two on Day 28 at 2 h post-dose for blood RNA assessments.

These blood samples were collected and processed as follows:

- One blood sample of approximately 10 mL was collected for IL-6, IL-8, TNF-α, CRP, fibrinogen, and SP-D analyses in plasma. Blood was collected in tubes containing lithium heparin, and was chilled in an ice bath and then centrifuged. Plasma samples were stored at –80°C, and then shipped to a central laboratory where they were stored at –80ºC until analysis.
- One blood sample of approximately 5 mL was collected into a tube containing 10 units heparin/mL for ex-vivo LPS-stimulated TNF-α determination. After incubation with LPS and processing, samples were stored at –80°C, and then shipped to a central laboratory where they were stored at –80ºC until analysis.
- Two blood samples of approximately 2.5 mL were collected into PAXgeneTM blood RNA tubes (PreAnalytiX GmbH, Hombrechtikon, Switzerland) for RNA analysis. The tubes were stored upright at room temperature (18°C to 25°C) for 2–72 h, then at –20°C for at least 24 hours, and then stored at –80°C until analysis.

## Sputum induction, collection and processing

To induce sputum, patients with FEV_1_ ≥0.8 L inhaled nebulised hypertonic saline at increasing concentrations (3%, then 4% and finally 5%), with FEV_1_ monitored for safety after each concentration. All patients wore a nose clip during this procedure, which was performed by qualified, trained personnel.

To evaluate PIP_3_, one aliquot of approximately 170 mg of sputum plug was transferred into a polypropylene tube and immediately frozen at –80ºC. It was shipped to the central laboratory on dry ice and stored at approximately –80ºC. The PIP_3_ peak area proportion was calculated from the HPLC mass spectrometer peak areas for PIP_3_ and PIP_2_ (PIP_3_ peak area/[PIP_3_ peak area + PIP_2_ peak area]), corrected for the corresponding deuterated internal standard, using established methodology [1].

For evaluation of target proteomics and biomarkers, sputum plugs were selected and homogenised with 1-fold Dulbecco's phosphate-buffered saline, and centrifuged to separate the cells from the fluid phase (supernatant). The fluid phase was removed, divided into four 200 µL aliquots and two 50 µL aliquots, and stored at –80ºC. The 50 µL aliquots were used for target proteomics assessments, with the 120 µL aliquot shipped to a central laboratory, and kept at approximately –80ºC for target biomarkers determination. The remaining cell pellet was treated with 0.2% dithiothreitol, before viability, total cell count (x10^6^ cells/g) and squamous cell contamination were determined. Cytospin slides were prepared and analysed centrally for differential cell counts (eosinophils, neutrophils, macrophages, lymphocytes). The remaining cellular material was processed for RNA extraction (to identify genes regulated by CHF6523).

## Proteomics

### Olink

For the proteomics evaluation, sputum samples were run on the Target 48 Cytokine panel, and the Target 96 Inflammation panel (Olink Protemics, Uppsala, Sweden). In the Target 48 Cytokine panel, 45 cytokines were quantified in terms of their absolute concentration. For the Target 96 Inflammation panel, 92 proteins involved in inflammation processes were evaluated, with the results expressed as Normalized Protein eXpression (NPX; the manufacturer’s arbitrary unit, in Log_2_ scale). The goal was to identify proteins that were significantly differentially expressed after dosing with CHF6523. In the statistical model, using sputum Target 96 and 48 data, change from pre-dose to post-dose expression were used as dependent variable, and patient, period, treatment and pre-dose expression as independent variable.

### TMT mass spectrometry

Sputum samples were weighed and solubilised with sodium dodecyl sulphate and dithiothreitol, and were incubated for 10 min at 95°C. First, quantification was performed by the Bradford Assay, with 50 μg of each sample digested with Trypsin following the S-trap micro protocol; 25 μg of each sample was dried, and reconstituted with TMT10plex (Thermo Scientific) following the manufacturer’s instructions. The 98 samples were divided into eleven TMT10plexes groups; 10 μg of each sample were combined, taking into account the peptide quantification, and after combining samples were desalted.

Samples were analysed with a Thermo Scientific Dionex Ultimate 3000 nano RSLC coupled to a Orbitrap Fusion Lumos mass spectrometer (Thermo Scientific) fitted with nanoESI source. Peptides were separated on a EasySpray (75 µm x 500 mm, 2 μm, 100Å) (Thermo Scientific) heated to 40°C at 300 nL/min. The mass spectrometer was operated in data-dependent acquisition (DDA) mode. The most abundant ions were selected for fragmentation by collision-induced dissociation (CID). For the MS3 analyses for TMT quantification, multiple fragment ions from the previous MS2 scan (SPS ions) were co- selected and fragmented by HCD.

Database searches were performed with Proteome Discoverer v2.4 software (Thermo Scientific) using Sequest HT search engine and UniProt Homo Sapiens (2023) and contaminants. The search was run against a targeted and decoy database to determine the false discovery rate (FDR). Search parameters included trypsin, allowing for two missed cleavage sites, carbamidomethyl in cysteine as static modification, TMT10plex peptide N-terminus and TMT6plex in K, methionine oxidation, and acetylation in protein N-terminus as dynamic modifications. The peptide mass tolerance was 10 ppm for MS1, 0.6 for MS2, and 20 ppm for MS3. Peptides with a q-value lower than 0.1 and FDR <1% were considered as positive identifications with a high confidence level. TMT reporter ions intensities were used for protein quantification.

Filtering was initially performed in ProteomeDiscoverer considering all protein groups with at least 2 unique peptides and FDR <0.1. An internal standard was split into 11 technical replicates for evaluating analytic correspondence between TMT sets, where little deviation was observed between sets, based on overall data dispersions and relative abundance density plots. Samples were excluded if deemed outliers based on overall protein distributions, number of total quantified proteins, coefficient of variation, interquartile ranges, density plots, projection in latent space through principle component analysis, or if they contained >10% missing values. Proteins were excluded if found in <90% of the samples. This resulted in a total of 413 proteins and 92 samples (and 11 technical replicate controls as internal standards).

Data was first normalised by mean-centring across TMT-batches using the technical replicate controls as references, and log_2_-transformed. To evaluate the impact of different normalisation methods in downstream statistics, from this point onwards we performed all subsequent statistical analyses in parallel on 4 datasets normalised through scikit-learn (quantile, robust scaler, powertransformer, standardisation) in addition to a comparison with an unnormalised dataset. All five datasets tended to display comparable distributions in the top 2 principle components, interquartile ranges, and quartiles 1–3, with the exception of three samples from the unnormalised dataset that tended to cluster separately from all other samples. Missing data was then imputed based on the NIPALS algorithm as implemented in the R Mixomics 6.17.26 [2,3].

Differential expression analysis was tested in two parallel approaches. First, an ANCOVA was performed on the change from baseline per patient per period, after excluding patients with no data in one of the visits (resulting in 20 patients). This was modelled by considering treatment as dependent variable, with patient and baseline abundance considered as covariates. In parallel, a Friedman test was also employed by modelling treatment as dependent variable, and patient as covariate. Second, a linear mixed effect model was applied by considering fixed effects of treatment and time (pre- and post-dose), and random effects on intercept for patient, i.e., abundance ~ treatment*time + (1 | subject). Contrasts for treatment*time effect were then evaluated for significance (post-dose minus pre-dose | CHF6523) minus (post-dose minus pre-dose | placebo). Both statistical approaches were substantially consistent across the five datasets, with only BPIFB1 showing as a statistically significant treatment effect (FDR <0.05 linear mixed effect modelling on robust scaled data; FDR <0.07 linear mixed effect modelling standardised data; FDR <0.15 ANCOVA on robust scaled change-from-baseline data; FDR <0.15 Friedman on quantile normalised data).

## Transcriptomics

PAXgene RNA blood samples and induced sputum samples collected in Trizol were analysed at Almac Diagnostic Services (Craigavon, Northern Ireland, UK). RNA extraction and quality assessment of the PAXGene blood and sputum samples were performed, prior to downstream next generation sequencing (NGS). To minimise any technical variability, samples were randomised into processing batches for RNA extraction and NGS library preparation.

### RNA extraction and quality control

RNA extraction from blood samples was performed using the Qiagen PAXgene Blood RNA Kit, with RNA isolated from the entire PAXgene blood RNA tube. Sputum samples were homogenised and processed using a standard phenol/chloroform extraction method using Trizol-LS reagent. This method combines a robust lysis/denaturant extraction with ethanol precipitation for recovery of high-quality total RNA. The crude RNA was further purified by incubation with DNaseI followed by filter purification using the RNeasy MinElute spin column to yield RNA in a volume of 12 µL.

After extraction, all RNA underwent quality assessment. PAXGene RNA samples were assessed using a combination of Nanodrop (quantity) and the Agilent Tapestation (integrity), and Sputum RNA samples were assessed using the Qubit RNA assay (quantity) and Agilent Tapestation (integrity). Nanodrop was used for quantity assessment of RNA extracted from sputum in order to preserve a sample for downstream library preparation input.

### RNA sequencing

Upon successful completion of the quality assessment, samples underwent library preparation using Kapa RNA Hyperprep with Riboerase kit. PAXGene Blood RNA samples were also treated with globin depletion oligos. Following library preparation, all generated libraries underwent quality assessment (quantification via Quant-iT dsDNA high-sensitivity assay, and fragment size assessment via the Agilent Tapestation) before being loaded on to a NovaSeq 6000 and sequenced with a total read length of 2x76bp, to a read budget of 50 million paired end reads (total 100 million reads) per sample. Processing control samples were included in the processing batch. Upon completion of sequencing, the quality of sequencing was assessed for the following parameters: clusters passing filter, Q30 scores, error rate, cluster density and read distribution.

### RNAseq quality control analysis

Raw sequence data in FASTQ format were subject to a number of quality control assessment steps hosted on the DNAnexus cloud platform. Basic sequencing metrics such as GC content were calculated from unaligned reads using FastQC. Potential ribosomal RNA (rRNA) contamination was assessed using the Burrows-Wheeler Aligner (BWA) and samtools flagstat to determine the number of reads aligning to a ribosomal RNA (rRNA) sequence database. Read alignment to the human reference genome GRCh38 was then performed using StarAlign, with outputs used to calculate post-alignment quality control (QC) metrics such as duplication rate, calculated using Picard MarkDuplicates and a housekeeping coverage metric using a custom script. This script considers 20 pre-selected genes demonstrated to achieve the most stable gene expression across previous in-house studies, and calculates median deduplicated read depth across these genes for each sample. Both pre- and post alignment QC metrics were then reviewed to check for potential problems originating from the sequencing run or starting library material.

The genes that were differentially expressed pre-dose were identified using an analysis of variance (ANOVA) that modelled pre-treatment/dose expression as dependent variable and treatment (reference: placebo), period and subject were independent variables.

The aim was to identify genes that were differentially expressed in response to treatment with CHF6523 from blood and sputum samples. To determine the effect of treatment on gene expression, ANCOVA and functional expression analysis were performed on the significantly differentially expressed genes from each comparison.

The ANCOVA model was specified as change from pre-dose to post-dose expression as dependent variable. The independent variables were treatment (reference: placebo), patient, period, and pre-dose expression.

Functional enrichment was performed on those differentially expressed gene lists that had at least 20 genes with FDR<0.05 using ToppFun enrichment on the ToppGene website (https://toppgene.cchmc.org/enrichment.jsp, accessed 05 May 2023 [4]). The background of the enrichment used were those genes that were available in the original datamatrix.

## Inclusion criteria

1. Written informed consent obtained prior to any study-related procedure;

2. Males and females aged ≥40 years;

3. A female was eligible to enter the study if of non-childbearing potential (WONCBP) i.e. physiologically incapable of becoming pregnant (e.g., postmenopausal, defined as being amenorrhoeic for ≥12 consecutive months without an alternative medical cause, confirmed by follicle-stimulating hormone levels, according to local laboratory ranges) or permanently sterilised (e.g. bilateral oophorectomy, hysterectomy or bilateral salpingectomy), or with non-fertile partner.

4. Male fulfilling one of the following criteria:

a. Non-pregnant women of childbearing potential (WOCBP) partners: they and/or their partner of childbearing potential were willing to use a highly effective birth control method in addition to the male condom from the signature of the informed consent and until 90 days after the follow-up visit. Subjects must not donate sperm during the study and for 90 days after the follow-up visit, or

b. Pregnant WOCBP partner: they were willing to use male contraception (condom) from the signature of the informed consent and until 90 days after the follow-up visit. Subjects must not donate sperm during the study and for 90 days after the follow-up visit, or

c. Non-fertile male subjects (including documented vasectomy) agreed to use condom, or

d. Male with partner not of childbearing potential (contraception is not required in this case).

5. Established diagnosis of COPD (according to GOLD guidelines, update 2019) at least 12 months prior to the screening visit;

6. Blood eosinophil count ≥150 cells/μL;

7. Smoking history of at least 10 pack-years. Current and ex- smokers were eligible. (Smoking cessation must have been at least 6 months prior to screening. Any smoking cessation therapy must have been completed at least 6 months prior to the screening visit);

8. Body-mass index in the range of 18–35 kg/m^2^;

9. Post-bronchodilator FEV_1_ ≥30% and ≤70% of the predicted value and post-bronchodilator FEV_1_/FVC ratio <0.70 measured 10–15 minutes after 400 µg (4 puffs x 100 µg) of salbutamol via pressurised metered dose inhaler;

10. With or without COPD exacerbation(s), i.e. resulting in the use of systemic (oral/IV/IM) corticosteroids and/or antibiotics or visit to an emergency department or hospitalisation, in the 24 months preceding the screening visit;

11. On maintenance triple therapy with ICS/LABA/LAMA combination within at least 6 months prior to screening visit;

12. Spontaneous sputum producer, i.e., able to produce an adequate induced sputum sample of at least 300 mg with a viability factor of not less than 70% (with less than 30% epithelial cells) at screening;

13. Symptomatic at screening, defined as having a COPD Assessment Test (CAT) score ≥10;

14. Able to be trained to correctly use the dry-powder inhalers and to generate sufficient peak inspiratory flow (at least 40 L/min) using the In-Check Dial device at screening and prior to randomisation;

15. Cooperative attitude and ability to perform the required outcome measurements (e.g., spirometry testing, sputum induction).

## Exclusion criteria

1. Women physiologically capable of becoming pregnant (i.e., WOCBP);

2. Pregnant or lactating female;

3. Current diagnosis of asthma;

4. Received or was planning to receive any type of vaccination within 3 weeks prior to screening visit;

5. COPD exacerbation resulting in the use of systemic (oral/IV/IM) corticosteroids and/or antibiotics or visit to an emergency department or hospitalisation or a lower respiratory tract infection within 6 weeks prior to the screening visit or during the period between screening and randomisation;

6. Receiving any maintenance therapy other than ICS/LABA/LAMA combination within 6 months prior to screening visit;

7. Receiving treatment with one or more prohibited medications (see below), and outside the allowed time windows;

8. Requiring long-term (at least 12 hours daily) oxygen therapy for chronic hypoxemia;

9. Participating in a pulmonary rehabilitation programme or completing such a programme within the 6 weeks prior to screening visit;

10. Known respiratory disorders other than COPD that in the investigator’s opinion would affect efficacy and safety evaluation or place the subject at risk;

11. Lung cancer or a history of lung cancer;

12. Active cancer or a history of cancer (other than lung) with less than 5 years disease-free survival time (whether or not there is evidence of local recurrence or metastases);

13. Known history of intolerance/hypersensitivity to any of the excipients/components contained in any of the formulations used in the trial;

14. Diagnosis of depression associated with suicidal ideation or behaviour or with a diagnosis of generalised anxiety disorder that in the investigator’s opinion would place the subject at risk;

15. Known history of clinically significant cardiovascular conditions such as, but not limited to, unstable or acute ischemic heart disease within one year prior to screening visit, NYHA Class III/IV heart failure, known history of sustained and non-sustained cardiac arrhythmias or history of atrial fibrillation diagnosed in the 6 months prior to screening visit and not controlled with therapy rate control strategy;

16. Clinically significant abnormal 12-lead ECG that, in the investigator’s opinion, would affect safety, pharmacokinetic or pharmacodynamic evaluation or place the subject at risk at screening or at randomisation visits;

17. Male subject with a QTcF >450 msec or female subject with a QTcF > 470 msec at screening or at randomisation visits;

18. History or symptoms of significant neurological disease including transient ischemic attack, stroke, seizure disorder or behavioural disturbances;

19. Unstable concurrent disease that might, in the judgement of the investigator, place the subject at undue risk or potentially compromise the results or interpretation of the study;

20. Clinically significant laboratory abnormalities indicating a significant or unstable concomitant disease that might, in the judgement of the investigator, place the subject at undue risk or potentially compromise the results or interpretation of the study, at screening visit;

21. Abnormal alanine aminotransferase (ALT) ≥2x upper limit of normal (ULN) and/or aspartate aminotransferase (AST) ≥2x ULN and/or bilirubin ≥1.5x ULN at screening visit;

22. Current or chronic history of liver disease, or known hepatic or biliary abnormalities (with the exception of Gilbert’s syndrome or asymptomatic gallstones);

23. Receiving treatment with any drug known to have a well-defined potential for hepatotoxicity (e.g. isoniazide, nimesulide, ketoconazole) within the 3 months prior to the screening visit or during the period between screening and randomisation;

24. Recent excessive weight loss that cannot be explained by the natural course of COPD or known background conditions;

25. Documented history of substance abuse or drug abuse within 12 months prior to screening visit or with a positive urine drug screen evaluated at screening or at randomisation visits;

26. Documented history of alcohol abuse within 12 months prior to screening visit or a positive alcohol breath test at screening or at randomisation visits;

27. Unsuitable veins for repeated venepuncture/cannulation;

28. Refuses to comply with study restriction;

29. Received any other investigational drug within the preceding 30 days (60 days for biologics), or a longer and more appropriate time as determined by the Investigator (e.g., approximately five half-lives of the previous investigational drug).

The use of short-acting muscarinic antagonists, alone or in combination with a short-acting β_2_-agonist, was not permitted from 12 h prior to screening and throughout the study. Use of the following was not permitted from the indicated time prior to screening and for the duration of the study: Oral, intravenous or intramuscular corticosteroids (6 weeks); depot corticosteroids (2 months); leukotriene modifiers (2 months); oral xanthine derivatives (2 months); phosphodiesterase-4 inhibitors, such as roflumilast (2 months). Any vaccination was allowed during the washout period between the two treatment periods, but at least 3 weeks had to elapse from the vaccination date to the first visit of the second period.

## Multi-omic data integration

Data pre-processing prior to integration was performed as follows. For blood and sputum RNA-seq, the top 30% most varying protein-coding genes were selected, with no subsequent filtering on Olink or mass spectrometry proteomics. All omic data (RNA-seq, mass spectrometry proteomics, Olink panels) were modelled through a mixed effect model with formula *abundance ~ 1 + treatment + time + treatment * time + (1 | patient)*, where *time* was either baseline or end of treatment, and the model residuals were then used for subsequent analysis. Plasma and sputum target biomarkers, PIP_3_, sputum and blood cell counts and blood chemistry numeric features were standardised, with categorical data not analysed further. Supervised data integration was performed through mixOmics (6.17.26, R 4.1.3) through a sparse partial least squares discriminant analysis, modelling four groups (CHF6523 baseline/post-treatment and placebo baseline/post-treatment). Model tuning was performed by scanning the number of latent variables and features per latent variable that minimise balanced error rate, for distance maximum, centroid and mahalinobis distances, through 5-fold cross-validation repeated 10 times. Areas under the receiving operating characteristic curves (AUROC) are reported for each comparison of a group vs the three remaining groups. All analyses were repeated within treatment by comparing post-treatment vs baseline for samples.

# Results

Table S1. Slow vital capacity, oscillometry, and symptoms results at Day 28 (pharmacodynamic set).

|  | **CHF6523 (N=31)** | **Placebo (N=35)** | **CHF6523 vs placebo** |
| --- | --- | --- | --- |
| Inspiratory capacity, L | –0.175  (–0.264, –0.085) (N=26) | –0.112  (–0.197, –0.027) (N=27) | –0.062  (–0.191, 0.066); p=0.320 |
| Vital capacity, L | –0.083  (–0.243, 0.076) (N=26) | –0.066  (–0.217, 0.086) (N=28) | –0.018  (–0.243, 0.207); p=0.871 |
| Resistance at 5 Hz, cmH_2_O/L/sec | |  |  |
| Inspiratory | 0.216  (0.066, 0.366) | 0.159  (0.024, 0.294) | 0.057  (–0.150, 0.264); p=0.574 |
| Expiratory | 0.112  (–0.149, 0.373) | 0.230  (–0.009, 0.470) | –0.118  (–0.485, 0.248); p=0.513 |
| Total | 0.172  (–0.013, 0.356) | 0.223  (0.055, 0.391) | –0.051  (–0.309, 0.207); p=0.686 |
| Resistance at 19 Hz, cmH_2_O/L/sec | |  |  |
| Inspiratory | 0.021  (–0.094, 0.136) | 0.107  (0.004, 0.210) | –0.086  (–0.245, 0.072); p=0.272 |
| Expiratory | –0.002  (–0.179, 0.176) | 0.008  (–0.156, 0.171) | –0.009  (–0.261, 0.242); p=0.939 |
| Total | 0.016  (–0.125, 0.156) | 0.055  (–0.072, 0.183) | –0.040  (–0.236, 0.157); p=0.683 |
| Resistance at 5 Hz minus resistance at 19 Hz, cmH_2_O/L/sec | | |  |
| Inspiratory | 0.198  (0.075, 0.321) | 0.051  (–0.061, 0.162) | 0.147  (–0.022, 0.317); p=0.085 |
| Expiratory | 0.112  (–0.080, 0.305) | 0.225  (0.050, 0.399) | –0.112  (–0.377, 0.153); p=0.392 |
| Total | 0.158  (0.018, 0.299) | 0.167  (0.039, 0.295) | –0.008  (–0.203, 0.186); p=0.929 |
| Reactance at 5 Hz, cmH_2_O/L/sec | |  |  |
| Inspiratory | –0.119  (–0.307, 0.068) | 0.021  (–0.149, 0.192) | –0.141  (–0.399, 0.117); p=0.272 |
| Expiratory | –0.336  (–0.801, 0.130) | –0.051  (–0.470, 0.367) | –0.284  (–0.919, 0.351); p=0.365 |
| Total | –0.263  (–0.623, 0.097) | –0.027  (–0.351, 0.298) | –0.237  (–0.729, 0.256); p=0.332 |
| Reactance at 19 Hz, cmH_2_O/L/sec | |  |  |
| Inspiratory | –0.113  (–0.257, 0.031) | –0.041  (–0.173, 0.090) | –0.072  (–0.270, 0.127); p=0.464 |
| Expiratory | –0.025  (–0.226, 0.177) | –0.104  (–0.287, 0.079) | 0.079  (–0.198, 0.357); p=0.563 |
| Total | –0.058  (–0.229, 0.112) | –0.086  (–0.240, 0.069) | 0.027  (–0.208, 0.262); p=0.814 |
| Tidal expiratory flow limitation, cmH_2_O/L/sec | 0.214  (–0.130, 0.557) | 0.102  (–0.211, 0.415) | 0.111  (–0.359, 0.582); p=0.630 |
| Breaths where expiratory flow limitation was present, % | 4.960  (–2.008, 11.928) | –1.522  (–7.992, 4.948) | 6.482  (–3.182, 16.145); p=0.179 |
| Resonance frequency, Hz | 0.167 (–0.960, 1.293) | 0.060 (–0.951, 1.071) | 0.106 (–1.434, 1.646); 0.888 |
| Respiratory rate, breaths/min | 0.268 (–0.359, 0.895) | –0.516 (–1.070, 0.038) | 0.784 (–0.078, 1.646); 0.073 |
| Tidal volume, L | 0.003  (–0.029, 0.036) | –0.008  (–0.038, 0.023) | 0.011  (–0.034, 0.056); p=0.620 |
| Mean inspiratory flow, L/sec | 0.023  (0.006, 0.041) | –0.006  (–0.021, 0.010) | 0.029  (0.006, 0.052); p=0.015 |
| COPD Assessment Test total score | –0.0  (–1.0, 0.9) (N=35) | –0.7  (–1.6, 0.3) (N=36) | 0.6  (–0.7, 2.0); p=0.340 |
| Rescue medication use | (N=37) | (N=38) |  |
| Days with no use, % | 49.0  (40.3, 57.7) | 54.7  (46.0, 63.3) | –5.7  (–17.9, 6.6); p=0.354 |
| Puffs/day | 1.69  (1.40, 1.99) | 1.42  (1.12, 1.71) | 0.28  (–0.14, 0.70); p=0.190 |

Data are adjusted mean change from baseline and adjusted mean difference (95% confidence interval), except the rescue medication data, which are adjusted mean and adjusted mean difference (95% confidence interval).

Table S2. Gene ontology (GO) biological processes, GO molecular functions, Wiki Pathways and Reactome pathways identified by functional enrichment analysis of the significant (Benjamini-Hochberg p<0.05) differentially expressed genes (DEGs) in sputum cells.

| **Category** | **ID** | **Name** | **p-value** | **False discovery rate** | **Hit count in query list** | **Hit count in genome** | **Hit in query list** |
| --- | --- | --- | --- | --- | --- | --- | --- |
| GO: Molecular Function | GO:0035663 | Toll-like receptor 2 binding | 1.47E-07 | 0.00004253 | 3 | 4 | TLR6, TLR10, TLR1 |
|  | GO:0071723 | lipopeptide binding | 5.97E-06 | 0.0008624 | 3 | 11 | TLR6, TLR10, TLR1 |
|  | GO:0061809 | NAD+ nucleotidase, cyclic ADP-ribose generating | 2E-05 | 0.001401 | 3 | 16 | TLR6, TLR10, TLR1 |
|  | GO:0050135 | NAD(P)+ nucleosidase activity | 2E-05 | 0.001401 | 3 | 16 | TLR6, TLR10, TLR1 |
|  | GO:0003953 | NAD+ nucleosidase activity | 2.42E-05 | 0.001401 | 3 | 17 | TLR6, TLR10, TLR1 |
|  | GO:0035325 | Toll-like receptor binding | 6.22E-05 | 0.002997 | 3 | 23 | TLR6, TLR10, TLR1 |
|  | GO:0016799 | hydrolase activity, hydrolysing N-glycosyl compounds | 0.000309 | 0.01276 | 3 | 39 | TLR6, TLR10, TLR1 |
| GO: Biological Process | GO:0071220 | cellular response to bacterial lipoprotein | 2.39E-06 | 0.001614 | 3 | 9 | TLR6, TLR10, TLR1 |
|  | GO:0071221 | cellular response to bacterial lipopeptide | 2.39E-06 | 0.001614 | 3 | 9 | TLR6, TLR10, TLR1 |
|  | GO:0070339 | response to bacterial lipopeptide | 2.39E-06 | 0.001614 | 3 | 9 | TLR6, TLR10, TLR1 |
| GO: Biological Process (continued) | GO:0032493 | response to bacterial lipoprotein | 3.41E-06 | 0.001725 | 3 | 10 | TLR6, TLR10, TLR1 |
|  | GO:0070340 | detection of bacterial lipopeptide | 2.87E-05 | 0.01084 | 2 | 3 | TLR6, TLR1 |
|  | GO:0006639 | acylglycerol metabolic process | 3.32E-05 | 0.01084 | 5 | 118 | PLB1, PLIN5, AVIL, SLC22A4, SORL1 |
|  | GO:0006638 | neutral lipid metabolic process | 3.75E-05 | 0.01084 | 5 | 121 | PLB1, PLIN5, AVIL, SLC22A4, SORL1 |
|  | GO:0019433 | triglyceride catabolic process | 4.89E-05 | 0.01158 | 3 | 23 | PLB1, PLIN5, SORL1 |
|  | GO:0010897 | negative regulation of triglyceride catabolic process | 5.72E-05 | 0.01158 | 2 | 4 | PLIN5, SORL1 |
|  | GO:0042494 | detection of bacterial lipoprotein | 5.72E-05 | 0.01158 | 2 | 4 | TLR6, TLR1 |
|  | GO:0046461 | neutral lipid catabolic process | 0.000147 | 0.0271 | 3 | 33 | PLB1, PLIN5, SORL1 |
|  | GO:0046464 | acylglycerol catabolic process | 0.000161 | 0.02719 | 3 | 34 | PLB1, PLIN5, SORL1 |
|  | GO:0006641 | triglyceride metabolic process | 0.000289 | 0.04503 | 4 | 102 | PLB1, PLIN5, SLC22A4, SORL1 |
| Pathway | M39478 | WP MYD88 DISTINCT INPUTOUTPUT PATHWAY  Source: WikiPathways | 1.69E-05 | 0.009827 | 3 | 18 | TLR6, TLR10, TLR1 |
|  | M27428 | REACTOME DISEASES OF IMMUNE SYSTEM  Source: Reactome pathways | 8.22E-05 | 0.02388 | 3 | 30 | TLR6, TLR10, TLR1 |

Figure S1. Multi-omic integration profiles for CHF6523 and placebo, indicating that only clinical parameters permit group distinction between baseline and end of treatment. Machine learning results for best models with CHF6523 (left) or placebo (right), including all data (top) or when excluding laboratory values (bottom), at baseline (orange) and end of treatment (blue). Each panel shows the projection of the top 2 latent variables (left), selected features resulting in lowest balanced error rate (centre), and classification AUROC (right). Colours indicate the group where features tend to display highest values. Model tuning performed through leave-one-out randomly repeated 10 times; all indicated transcripts (RNA-seq) are from sputum.


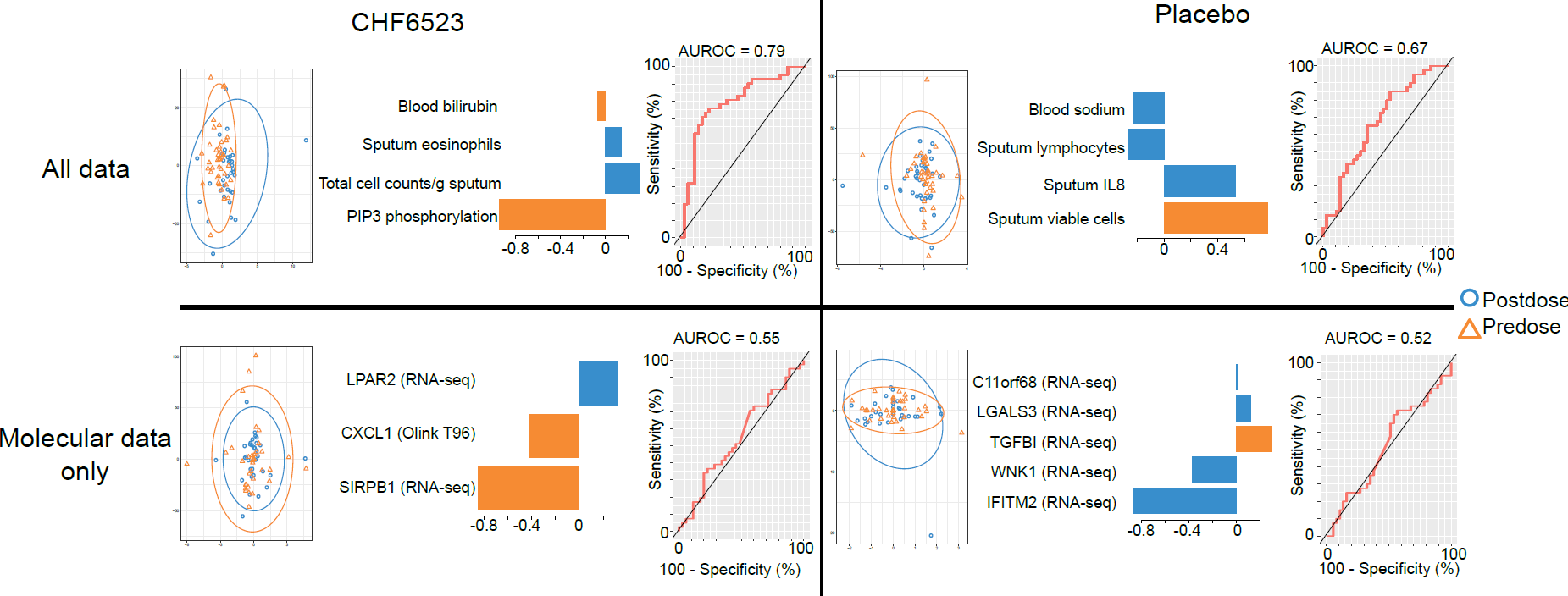


# References

1. Clark J, Anderson KE, Juvin V, Smith TS, Karpe F, Wakelam MJO, et al. Quantification of PtdInsP3 molecular species in cells and tissues by mass spectrometry. Nat Methods 2011;8:267–72. doi:10.1038/NMETH.1564

2. Wold H. Estimation of principal components and related models by iterative least squares. In: Krishnaiah P, editor. Multivariate Analysis 1966. p. 391–420.

3. Wold H. Path models with latent variables: The NIPALS approach. In: Blalock H, Aganbegian A, Borodkin F, Boudon R, Capecchi V, editors. Quantitative Sociology: International perspectives on mathematical and statistical model building 1975. p. 307–57.

4. Chen J, Bardes EE, Aronow BJ, Jegga AG. ToppGene Suite for gene list enrichment analysis and candidate gene prioritization. Nucleic Acids Res 2009;37:W305–11. doi:10.1093/nar/gkp427
